# Establishing a Cohort at High Risk of HIV Infection in South Africa: Challenges and Experiences of the CAPRISA 002 Acute Infection Study

**DOI:** 10.1371/journal.pone.0001954

**Published:** 2008-04-16

**Authors:** Francois van Loggerenberg, Koleka Mlisana, Carolyn Williamson, Sara C. Auld, Lynn Morris, Clive M. Gray, Quarraisha Abdool Karim, Anneke Grobler, Nomampondo Barnabas, Itua Iriogbe, Salim S. Abdool Karim, for the CAPRISA 002 Acute Infection Study Team

**Affiliations:** 1 Centre for the AIDS Programme of Research in South Africa (CAPRISA), University of KwaZulu-Natal, Durban, South Africa; 2 Institute for of Infectious Disease and Molecular Medicine, University of Cape Town, Cape Town, South Africa; 3 Columbia University, New York, New York, United States of America; 4 National Institute for Communicable Diseases, Johannesburg, South Africa; Instituto de Medicina Tropical Alexander Von Humboldt, Peru

## Abstract

**Objectives:**

To describe the baseline demographic data, clinical characteristics and HIV-incidence rates of a cohort at high risk for HIV infection in South Africa as well as the challenges experienced in establishing and maintaining the cohort.

**Methodology/Principle Findings:**

Between August 2004 and May 2005 a cohort of HIV-uninfected women was established for the CAPRISA 002 Acute Infection Study, a natural history study of HIV-1 subtype C infection. Volunteers were identified through peer-outreach. The cohort was followed monthly to determine HIV infection rates and clinical presentation of early HIV infection. Risk reduction counselling and male and female condoms were provided. After screening 775 individuals, a cohort of 245 uninfected high-risk women was established. HIV-prevalence at screening was 59.6% (95% CI: 55.9% to 62.8%) posing a challenge in accruing HIV-uninfected women. The majority of women (78.8%) were self-identified as sex-workers with a median of 2 clients per day. Most women (95%) reported more than one casual sexual partner in the previous 3 months (excluding clients) and 58.8% reported condom use in their last sexual encounter. Based on laboratory testing, 62.0% had a sexually transmitted infection at baseline. During 390 person-years of follow-up, 28 infections occurred yielding seroincidence rate of 7.2 (95% CI: 4.5 to 9.8) per 100 person-years. Despite the high mobility of this sex worker cohort retention rate after 2 years was 86.1%. High co-morbidity created challenges for ancillary care provision, both in terms of human and financial resources.

**Conclusions/Significance:**

Challenges experienced were high baseline HIV-prevalence, lower than anticipated HIV-incidence and difficulties retaining participants. Despite challenges, we have successfully accrued this cohort of HIV-uninfected women with favourable retention, enabling us to study the natural history of HIV-1 during acute HIV-infection. Our experiences provide lessons for others establishing similar cohorts, which will be key for advancing the vaccine and prevention research agenda in resource-constrained settings.

## Introduction

As we move into the third decade of the AIDS epidemic, the need to increase and improve the research being conducted in some of the hardest hit areas of the developing world has become increasingly clear. These regions pose unique challenges for scientific research as they are typically resource-poor settings with minimal existing infrastructure and populations that are not well integrated into a formal health care system. In order to conduct effective research into the HIV epidemic in southern Africa, it is critical to improve our ability to maintain longitudinal cohorts in the face of significant social, cultural, and logistical hurdles.

In order to determine which host and viral factors during the acute and early phases of HIV-1 infection have a significant impact on the subsequent course of disease, the Centre for the AIDS Programme of Research in South Africa (CAPRISA) initiated the CAPRISA 002 Acute Infection Study. This study will characterize HIV-1 subtype C viral load set point in heterosexual infection in South Africa, the role of specific T cell immune responses in the control of replication, and the relationship between viral set point, CD4+ T cell count trajectory and disease progression.

The design of this study overcomes several limitations of previous acute infection studies. While several earlier studies used a large window period for identification of acute infection [1]–[5], others relied on patient recall of symptoms associated with acute retroviral syndrome [6]. Still other studies focused on one particular aspect of acute HIV infection, such as clinical signs and symptoms [7], without placing the clinical presentation in the context of virological and immunological responses. Furthermore, the majority of these studies were conducted in developed countries where both the context for the research and the viral subtypes are markedly different from those that are found in southern Africa and the developing world. The only prospective acute infection study that was conducted in Africa identified acute infections within a period averaging 1.1 months but was limited by its focus on clinical manifestations of acute infection [8]. As a prospective observational cohort study, this study will also provide a methodology for setting up and managing an observational cohort requiring frequent visits over an extended period of time against within a very challenging, resource-constrained environment with participants who tend to be highly mobile and difficult to contact by conventional means.

To our knowledge, this acute infection study will be the first in southern Africa to document acute infection in a prospective cohort with extensive follow-up on the natural history of HIV-1 subtype C infection. The aim of this paper is to provide a preliminary description of the demographic and clinical characteristics of the cohort of HIV-uninfected participants, the challenges in administering the cohort, and some of the successful strategies employed to overcome these obstacles. This paper provides important information with respect to the establishment of high-risk cohorts for acute HIV infection and other prospective observational studies. Establishment of cohorts in developing country setting have unique challenges which differ between locations and populations. Our cohort consists primarily of high risk HIV-uninfected women working as female sex workers, recruited from a large urban area. This cohort is of high potential impact as forms part of a larger acute infection study which measures virological, immunological and clinical events in acute and early infection. Results from this study are likely to provide valuable information to inform vaccine trials, as well as understanding host, immunological and virological correlates of disease progression in subtype C infection. This paper provides important information with respect to the establishment of high-risk cohorts for acute HIV infection and other prospective observational studies.

## Materials and Methods

The CAPRISA 002 Acute Infection Study is being conducted at the Doris Duke Medical Research Institute (DDMRI) at the Nelson R Mandela School of Medicine of the University of KwaZulu-Natal in Durban, South Africa. The goal of this study is to identify acute HIV infection and, by prospectively following participants with acute infection, provide thorough information on the natural history of HIV-1 subtype C infection.

### Cohort Development

Building on participatory research methods developed for earlier work in similar cohorts [9]–[11], a network of ten community liaison persons (CLPs) was established prior to initiation of the study to assist with study recruitment and retention efforts. Recruitment was based upon word-of-mouth and site visits by the CLPs. Initially we recruited directly within 5 kilometres of the clinic site, but CLPs did bring women in from as far as around 45 kilometres (approximately 3 to 28 miles). Most of the recruitment sites were associated with transport links into the city, and so participants were able to access the existing system of public transport. Participants received reimbursement for time, effort and their transport expenses when they visited the clinic. We limited our recruitment radius so that no woman would have to use her own money. Women from the community who were at least 18 years old and either self-identified as female sex workers (FSWs) or reported having had at least three partners in the 3 months prior to recruitment were screened for participation in the study. Young women in urban South Africa are already at high risk for HIV infection [12], and our goal was to identify women who would theoretically be at the greatest risk of HIV acquisition. Women who were pregnant at the time of screening or who planned to travel away from the study site for more than 3 months were excluded from participation.

The screening procedure for identifying HIV-negative women consisted of voluntary counselling and testing (VCT) followed by a blood collection for rapid HIV antibody testing and urine collection for pregnancy testing. If the first antibody test (Determine: Abbott Laboratories, Tokyo, Japan) was negative, the participant was enrolled into the HIV-negative cohort. If the first antibody test was positive, a second rapid antibody test (Capillus; Trinity Biotech, Jamestown, NY, USA) was administered. Those with a two positive antibody tests were referred for HIV follow-up care. Those with discordant antibody results were given a confirmatory HIV enzyme immunoassay (EIA) (BEP 2000; Dade Behring, Marburg, Germany) and were either enrolled into the HIV-negative cohort or referred for HIV follow-up care on the basis of those results.

In order to maximize participant retention, the study visit schedule was thoroughly explained during the informed consent process and was re-emphasized at each study visit. Detailed locator information was collected and was reviewed at each subsequent visit and a secure participant-tracking database was established to facilitate visit scheduling and prompt follow-up for missed visits. Additionally, participants were compensated (100 South African Rand, ∼$14USD, as approved by the ethics committee) for their transport to the study site and for their time at the clinic.

To improve communication with the cohort, a web-based short message service (SMS) is used to send pre-approved cell phone text messages to willing participants. This system is used to remind participants who generally do not have access to fixed-line telephone services about their study appointments. The service is cost effective (0.33 South African Rand per message) and also provides an electronic record of all messages sent and whether or not they are received.

If a participant misses a visit, the clinic nurse administrator attempts to call a participant for the first 3 days after the clinic visit. A SMS with the clinic contact number is also sent out on the second and third days. If these attempts at contact are unsuccessful by the second week, the study community liaison officer attempts person-to-person contact by travelling to the participant's documented contact site as well as to the site from which the participant was originally recruited.

In addition to the methods above, the study staff and community liaisons regularly communicate with the study community at large to increase awareness of HIV/AIDS and explain the purpose of HIV prevention research and the importance of completing research study visits. A Community Research Support Group (CRSG) comprised of the CLPs meets bi-monthly with study representatives to discuss and provide feedback on the progress of the study and to discuss challenges faced by the study team with regards to community and participant relations.

### Participant Protection

The study protocol and informed consent documents were reviewed by the local ethics committees of the University of KwaZulu-Natal, the University of Cape Town, the University of the Witwatersrand in Johannesburg, and by the Prevention Sciences Review Committee (PSRC) of the Division of AIDS (DAIDS, National Institutes of Health, U.S.A.). The consent forms were translated into isiZulu and written informed consent is obtained at each stage of the study (screening, enrolment into HIV-negative cohort, enrolment into acute infection phase, and for sample storage). All women screened for participation, whether ultimately enrolled into the study cohort or not, receive HIV pre- and post-test counselling, risk reduction counselling, male and female condoms, access to clinical care, and treatment for sexually transmitted infections (STIs). HIV/STI risk reduction counselling, condom provision, and prevention education supplies are administered at each subsequent study visit. One-on-one counselling was provided by a non-governmental organization which offered the same service in the public sector in the region, to be consistent with the services in the region. Overcoming one of the key challenges to doing HIV-related work in this context [11], participants who were HIV infected during the study were referred to the CAPRISA Antiretroviral Treatment (CAT) programme where they were offered ongoing care, and treatment for HIV when clinically eligible.

### Study Visits and Procedures

Once enrolled into the HIV-negative observational cohort, the participants underwent a baseline evaluation that included: a HIV behaviour risk assessment, a clinical evaluation, blood collection for routine laboratory assessment and HIV status, and a specimen collection for sexually transmitted infection (STI) diagnosis.

Following the baseline evaluation, participants in the HIV-negative cohort attend monthly follow-up evaluations for a maximum of 24 months. The monthly evaluations consist of a clinical evaluation, two HIV antibody rapid tests, HIV EIA, and pooled HIV-1 RNA testing (Amplicor v1.5: Roche Diagnostics, Rotkreuz, Switzerland). A urine dipstick is done on clinical suspicion of pregnancy, or if requested by the participant. In addition, a routine laboratory assessment, urinalysis, and STI screening are performed at 6-monthly intervals.

Women from the HIV-negative cohort are diagnosed with acute HIV infection by (1) the detection of HIV-1 antibodies within 5 months of a previously negative HIV-1 test; or (2) evidence of HIV-1 viral replication in the absence of HIV-1 antibodies. While women in this cohort were screened monthly allowing for rapid identification of acute infection, a 5 months window period was allowed to enable to recruit from other prevention cohorts where follow-up was only quarterly. Time of infection is defined as the mid-point between the last HIV antibody negative test and the first HIV antibody positive test; or if a positive RNA PCR assay is available on the same date as a negative HIV EIA, the date of HIV infection is estimated at 14 days prior to the first positive RNA PCR assay.

### Staff Training and Infrastructure Development

Given the extended scope and longitudinal nature of this study, CAPRISA was required to expand the size of the staff and the ability of the organization to manage a large cohort. After hiring additional clinicians, nurses, counsellors, and laboratory personnel, all team members underwent extensive training in both good clinical practice (GCP) and in the particulars of this study with respect to the case report forms (CRFs) for documentation, the quality control plan, and Human Subjects Protections (HSP). Furthermore, the capacity of the laboratory had to be increased to handle the large quantity of specimens related to the study.

### Data Management and Statistical Analysis

All data is entered onto case report forms (CRFs) at the study sites and faxed into the CAPRISA Data Management Centre, using the DataFax system (Clinical DataFax Systems Inc., Ontario, Canada). This capability did not previously exist at site, and had to be implemented in order to collect the data for this study. Source documents and original CRFs are maintained on site while an electronic version of the CRF is maintained by the Data Management Centre. Quality assurance and quality control are maintained and checked at specified intervals. All analyses are conducted using the SAS statistical package version 9.1 (SAS Institute, Cary, NC, U.S.A.).

## Results

### Establishing the Cohort

**Figure 1:**
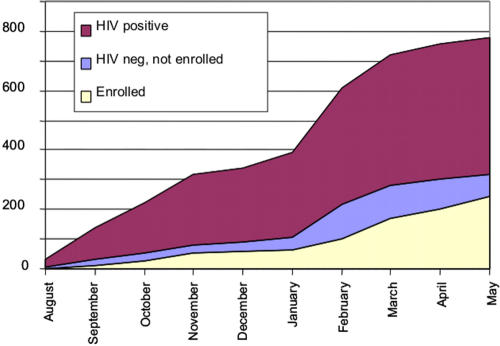
Cumulative HIV serostatus of screening participants over time.

Between August 2004 and May 2005, 775 women who were classified as high-risk either by self-identification as sex workers or self-report of more than three sexual partners in the previous 3 months were screened for participation in the observational cohort. Recruitment was done at known FSW sites in the city, therefore the majority of these women (n = 193, 78.8%) were self-identified as sex workers. The cumulative number of women screened and enrolled over the entire 10-month period can be seen in Figure 1.

**Figure 2:**
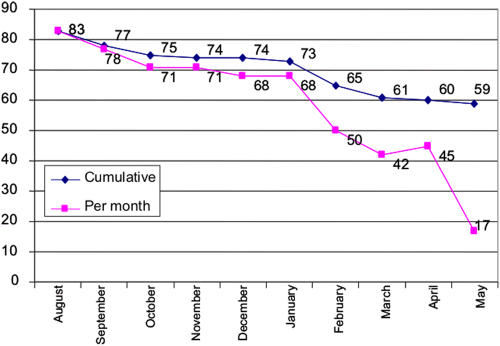
HIV prevalence in participants screened for enrollment into Phase I of the study over time, both cumulative and per month.

Of the 775 women screened, 59.6% (95% CI 55.9%–62.8%) were found to be HIV-positive. The mean ages of the women who were screened and found to be HIV-positive and HIV negative were 29.3 years (range 16–58) and 34.2 years (range 18–58), respectively (p<0.001). The HIV prevalence among the screening participants changed dramatically over time from 83% in the first month of screening to 17% in the last month of screening (Figure 2).

Of the 313 women who were HIV-negative, 245 HIV-negative women were eligible and agreed to participate in the HIV-negative cohort. Exclusion criteria for women who were uninfected at screening were as follows: less than three sexual partners in the previous 3 months (n = 22), women who were pregnant at the time of screening (n = 16), women who planned to relocate (n = 4), women younger than 18 years (n = 3), and women who were afraid of the testing procedures (n = 2). There were twenty-one women who were eligible following screening but never returned for enrolment into the HIV-negative cohort.

The demographic characteristics of the 245 women enrolled in the HIV-negative cohort are presented in Table 1. The mean age of the overall cohort was 34.3 years (range 18–58). Approximately 42.0% of the cohort had 11 or 12 years of education or higher, 33.5% had between 8 and 10 years of education, and 24.5% had an education level less than 8 years.

**Table 1:** Demographic characteristics of the HIV-negative observational cohort.

	Number (N = 245)	Percent		Number (N = 244)	Percent
**Race/ethnicity**			**Marital status**		
Black	241	98.4	Single, no steady partner	10	4.1
White	3	1.2	Married	16	6.5
Indian	1	0.4	Divorced	3	1.2
			Widowed	3	1.2
**Education**			Stable partner	75	30.6
> Grade 11	103	42.0	Multiple partners	135	55.1
Grade 8–10	82	33.5	Separated	2	0.8
< Grade 8	60	24.5			

(Note: where women refused to answer certain questions, the category total is less than the cohort total of 245.)

The numbers of steady and casual partners reported by the women are listed in Table 2. Steady partners were defined as someone seen “most of the time, often over a period of time” while a casual partner was someone the women saw only occasionally or even only once (but not a client in the case of the FSWs).

**Table 2:** Sexual partner history for the 245 women in the HIV-negative observational cohort.

	Partners in the previous 3 months				Lifetime partners			
	Steady		Casual		Steady		Casual	
# of partners reported	Number	Percent	Number	Percent	Number	Percent	Number	Percent
***0***	7	2.9	4	1.7	2	0.8	0	0
***1***	158	65.6	14	5.9	9	3.7	0	0
***2–3***	62	25.7	166	69.5	42	17.4	32	13.4
***4–6***	8	3.3	33	13.8	80	33.1	31	13.0
***7–12***	1	0.4	3	1.3	20	8.3	17	7.1
***>12***	0	0	0	0	7	2.9	2	0.8
***Too many to remember***	5	2.1	19	7.9	82	33.9	156	65.5
***Total***	**241**	**100**	**239**	**100**	**242**	**100**	**238**	**100**

(Note: where women refused to answer certain questions, the category total is less than the cohort total of 245.)

All of the women in the cohort have engaged in peno-vaginal intercourse; 34.6% and 25.4% of the cohort have engaged in anal and oral sex respectively. The mean age at sexual debut was 17 years (range 12–26) and at the time of study enrolment the mean number of days since last sexual contact was 4.8 (range 1–45). While 58.8% of participants indicated that a condom was used at last sexual encounter, 13.9% and 34.3% of the women indicated that they were never able to insist on a condom being used with casual and steady partners respectively (Table 3). Additionally, a high proportion of the women (75.7%) provided economic support to adults (mean 0.9, range 0–7) or children (mean 2.8, range 0–14).

**Table 3:** Ease of condom use with casual and steady partners, showing a trend towards ease of condom use with casual partners, and lower and inconsistent condom use with steady partners (Fisher's exact test; p<0.001).

Ease of Condom Use	Casual (% and n)	Steady (% and n)
Never	13.9% (34)	34.3% (84)
Sometimes	32.2% (79)	43.3% (106)
Every Time	53.9% (132)	20.4% (50)

Among the women who self-identified as FSWs, the median length of sex work was 3 years (range 1 month–31 years) and the median age at which the women began sex work was 26 years (range 14–53 years). The median number of days performing sex work per week was 3 days (range 1–7 days) with a median of 2 clients see per day (range 1–10), with a median of 2 clients (range 0–30) seen in the previous week.

### Maintaining the Cohort

Enrolment for the Phase 1 HIV-negative cohort began in September 2004 and the last participant was terminated in May 2007. The retention rate for the HIV-negative cohort was 86.1%. Despite the high mobility and low socioeconomic status of this population, only 13 (5.3%) of the HIV-negative cohort were terminated because they could not be traced. Other reasons for termination included participant relocation (n = 13), inability to adhere to the study schedule (n = 4), participant withdrew consent (n = 4), and death due to causes unrelated to study participation (n = 5).

As part of the study protocol, the women in the cohort received regular clinical examinations. Key baseline characteristics are summarized in Table 4. On enrolment into the cohort, the HIV-negative cohort had a mean body mass index (BMI) of 31.0 kg/m^2^. Of the 245 participants, 121 (49.4%) had a BMI greater than 30 kg/m^2^ (defined as obese) and a further 58 (23.7%) were overweight with a BMI between 25 and 30 kg/m^2^. Sixty-two participants (25.3%) had a normal BMI between 18.5 and 25 kg/m^2^ and only five participants (2.0%) were underweight with a BMI less than 18.5 kg/m^2^. Despite the high prevalence of obesity in this cohort, hypertension was not common. The mean systolic blood pressure was 117 mmHg and the mean diastolic pressure was 76 mmHg. Only 29 (11.8%) of the women had a systolic pressure greater than 140 mmHg or a diastolic pressure greater than 90 mmHg. Moderate anaemia was relatively common in this population and 48 (19.7%) of the women had a haemoglobin less than 12 g/dL at study enrolment.

**Table 4:** Baseline medical characteristics of the HIV-negative cohort (n = 245).

	Mean (SD)	Range
Blood pressure (mmHg)		
Systolic	117 (15)	90–200
Diastolic	76 (10)	55–110
Body mass index (kg/m^2^)	31.0 (7.8)	17.2–54.5
Hemoglobin (g/dL)	12.7 (1.3)	7.7–16.1
Hematocrit (%)	37.3 (0.3)	25–46

On enrolment into the cohort and at 6-monthly intervals, these women underwent STI screenings for *Trichomonas vaginalis*, *Neisseria gonorrheae*, *Chlamydia trachomatis*, *Mycoplasma genitalium*, syphilis, and Herpes simplex virus-2. Overall, 29.4% of women were infected with an STI at baseline. This percentage increases to 62.0% if bacterial vaginosis is included. Further, while none of the women were pregnant on enrolment, there were 32 pregnancies in the cohort for an incidence of 8.5 per 100 person-years (95% CI 5.6–11.5). During this same period, there have been 5 deaths unrelated to study participation. Causes of death include: stab wound (1), clinically reported as idiopathic thrombocytopenia (1), and unknown (3).

Finally, after 4784 monthly visits by 245 participants in this HIV-negative cohort, we identified 28 acute HIV infections. Twenty of the acute infections were among women who had self-identified as female sex workers and 8 of the acute infections were among women who had reported more than three partners in the preceding 3 months. The annual seroincidence after 390 person years was 7.2 per 100 person-years (95% CI 4.5–9.8).

**Figure 3:**
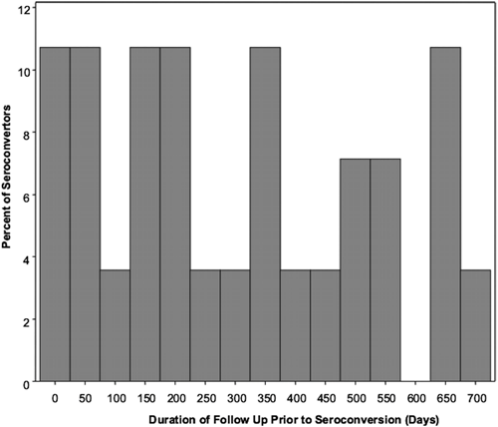
Percentage of acute HIV infections as a function of time in follow up (in days), showing a decrease in acute infections over time of follow up for the study.

We investigated if the number of new HIV infections reduced over time, which could be due to women who were most at risk of getting infected seroconverting early during follow-up or women reducing their risky behaviour over time and in response to repeated risk-reduction counselling. There was a significant trend over time in the rate at which women seroconverted (p-value = 0.0283), and this is graphically illustrated in Figure 3.

## Discussion

This paper describes the recruiting strategies, screening methods, enrolment procedures and retention techniques for a cohort of high-risk South African women, consisting largely urban female sex workers, a typically transient and mobile population that formed the basis for the CAPRISA 002 Acute HIV Infection study. We encountered numerous challenges over the course of establishing and administering the cohort and this paper aims to describe our strategies for overcoming these challenges.

The first challenge was defining the inclusion and exclusion criteria for the cohort. The study design called for uninfected yet “high risk” women so that we could theoretically maximize the number of acute infections we were able to identify. As we began recruitment, we were confronted with cultural and social barriers to delineations within this population. While many women were involved in transactional sex of some form, they were often reluctant to identify themselves as FSWs. Transactional sex for money or goods with non-regular partners is common in this region and is often not explicitly described as commercial sex work [13]. Once these cultural taboos became apparent, our study staff made a concerted effort to ensure that transactional sex was asked about in an open and permissive manner. Close work with the CLPs, some of whom were self-identified FSWs, enabled the research team to systematize a non-judgmental approach. Future work in similar cohorts needs to take account of the fact that terms such as ‘sex worker’ or ‘client’ may be inappropriate, and that broader definitions that include multiple partners who provide material needs in exchange for sex should be included in behavioural risk assessments.

The screening was made more difficult because of the high prevalence of HIV (59.6%) in the screening population. This is an extremely high prevalence rate, even when compared with the reported prevalence rates of 13.3% for South African women, 16.5% for the highly-burdened KwaZulu-Natal province, and 33.3% for South African women between the ages of 25 and 29 years [14]. The change in HIV prevalence rates over the course of screening was quite dramatic – from 83% to 17% – with the majority of uninfected participants identified in the final quarter of recruitment. The decline in prevalence over the course of screening could reflect a desire of women who were most concerned about their status to come first to the clinic for HIV testing, or for women who were most active in sex work to come forward first. Regardless, this discrepancy highlights the fact that cross-sectional surveys of seroprevalence can be a highly variable, or even unreliable, indicator of true prevalence and underscores the benefits of having an extended screening period with adequate numbers of screening participants. Enrolling participants over time from different populations with different risk profiles could bias the results of many studies. This variation is particularly significant for prevention studies where care should be taken to design the randomization process to equally distribute these differences with respect to time and intervention. For example, it is important to use blocked randomization to ensure that equal numbers of participants are enrolled at each stage of the trial into the different treatment arms. Accordingly, any trends over time in these studies must be interpreted with caution as these trends could be due to baseline differences in the participants.

In our study there was a significant age difference between the HIV-positive women who were screened (mean 29.3 years) and the HIV-negative women (mean 34.3 years) who were enrolled into the study cohort. This difference may be a sign of the changing demographics of the HIV epidemic among high-risk women in South Africa and supports the view that the epidemic is being most acutely experienced by younger South African women. On the other hand, lower HIV infection susceptibility, potentially (but controversially) associated with factors such as HLA alleles, partner-specific alloimmunity, reduced CD4+ T cell susceptibility, variability of cellular proteins involved in HIV-1 replication, host antiviral cellular proteins, or some related host genetic factor [15]–[23], may explain why some of the women have remained negative despite high-risk exposures and therefore remain uninfected although significantly older. That is, the women who are susceptible to infection are infected at an early age, leaving the less susceptible women as the core of the HIV-negative cohort. This trend is also supported by our finding that women who remain the in cohort are statistically less likely to seroconvert as follow-up progresses. Further research has been initiated to examine some of these factors in the HIV-negative, high-risk women that remain uninfected at the end of their study follow-up.

Another major challenge of working with this cohort of HIV-1 negative women has been to reconcile the study goals of identifying acute infections with the ethical imperative to aggressively promote prevention. Prevention efforts in this cohort included the provision of male and female condoms along with monthly HIV risk-reduction counselling. In spite of these efforts, the high rate of pregnancies and STIs in this cohort underscores the difficulty of prevention promotion in this population. Similar to vaccine or microbicide studies that intensively promote prevention, our experience is that some women will remain at risk for HIV infection in spite of the best efforts of the research team.

Retention rates for the HIV-negative observational cohort have been extremely successful with close to 86% retention over the duration of follow-up. Given the highly mobile nature of this population, these rates are high and bode well for future microbicide and vaccine studies which could have even greater retention rates in light of the perceived benefits of the intervention to the participants. We believe that this success underscores the value of having a dedicated network of community liaisons and of making a coherent and concerted effort to engage the community on a structural level. Making use of all available contact mechanisms and channels, especially the cell phones and short message system technology, proved to be essential for maintaining follow-up in this highly mobile cohort in which fixed-line telecommunications access is not common. We have established the feasibility and utility of using these services to both remind patients about their study visits and to follow up with missed visits.

Additionally, while the study's CLP network was initially intended as an additional measure to assist with participant tracking and retention, we found that the designated CLPs were ultimately only effective in tracing women with whom they had pre-existing relationship or acquaintance. As the study progressed, the study staff would increasingly rely on friends and relatives to relay messages to participants. Other factors that have undoubtedly contributed to the high retention rates include the monetary compensation for study visits, the regular counselling sessions and subsequent relationships formed with the counsellors, the distribution of condoms, and the knowledge that participants will be referred for STI treatment and other medical services as necessary.

We have also presented here an overview of the demographic, behavioural, and clinical characteristics of this cohort. In reviewing the clinical profile of these women, while they are a relatively young population and would thought to be healthy, they have a number of comorbidities. The rates of hypertension (11.8%) are lower than reported rates for South Africa of 21.1% [24] but the cohort tended to be overweight or obese with a relatively high prevalence of anemia. The presence of these comorbidities will be important for the clinical management of these women and will need to be considered among HIV-infected South African women. They also have high rates of STIs and pregnancy despite intensive counselling and ready availability of prevention materials. Lastly, the non-study related deaths of five of the participants reveals that even HIV-uninfected women living in urban South Africa are at high-risk of death. As we continue to accrue longitudinal data on this cohort, we anticipate that these baseline characteristics will be expanded on, and specific results on anaemia during acute infection have been published [25].

The behavioural data provides an overview of the sexual history and potential exposures of this theoretically high-risk cohort, particularly the FSWs. A previous survey of sex workers in the KwaZulu-Natal province of South Africa found that women working at truck stops averaged 22 sex acts per week, with coitus occurring between two to ten times in a 24 hour period [9]. This earlier survey also found that peno-vaginal sex was the only practice reported, with strong cultural sanctions against anal and oral sex. The sex workers in this urban cohort appear to have customers with whom they engage in fewer acts but see more regularly, averaging just three clients per week, and are also engaging in anal and oral sex. Consequently, they have a very different risk profile than what had been anticipated based on the prior studies. This difference in risk behaviour has likely impacted the HIV seroincidence in this cohort. Data are being analysed to determine the specific behavioural risk factors for HIV infection in this cohort.

The perceived ease of condom use in this cohort shows that women are significantly more likely to feel that they will be able to use condoms with casual partners than with their steady partners. This difference may explain why condom use has not led to a reduction in HIV risk as these women are still vulnerable due to inconsistent condom use with their steady partners. This observation suggests that condom use may be associated with perceptions of trust; whatever the case, condom use is inconsistent at best in this population. The behavioural risk data collected at baseline will be analysed after the full 24 month follow-up period to see which factors are most predictive of HIV acquisition in this cohort.

Based on previous data, the projected annual sero-incidence for this high-risk population was 18.2% [9], [12]. The actual seroincidence in this cohort has been 7.2% which is a little higher than the national seroincidence estimates of 6.3% for all South African women between the ages of 15 and 49 [14]. The risk of HIV acquisition in the context of the maturing South African HIV epidemic seems to be highly generalized. Hence, our designations of “high risk” are seemingly less relevant in retrospect and perhaps need not be considered as rigorously when designing future studies. Furthermore, unmeasured susceptibility factors may have disproportionately influenced the observed infection rate in this cohort of highly exposed individuals, where the women who were more susceptible had acquired HIV prior to screening for this study. Furthermore, our data support the notion that high-risk cohorts may yield fewer seroconversions over time, and that this attrition should be factored into the design of such cohorts. Women who remain in the cohort at the end of the follow-up, may represent a group enriched with host genetic resistance factors, and this is currently being explored as this group of woman has been recruited into a highly exposed persistently negative cohort for additional study.

### Conclusion and Significance

To the best of our knowledge, this is the first prospective cohort to be assembled in southern Africa for a comprehensive analysis of the behavioural, clinical, and immunological characteristics associated with acute HIV infection. The methods, as well the challenges, of recruiting and successfully retaining a HIV-negative cohort have been described and can provide guidance for others wanting to assemble similar cohorts. Given the characteristics of our cohort, these experiences are particularly relevant to the recruitment and retention of cohorts of urban female sex workers in South Africa for HIV prevention and pathogenesis research. The changing nature of the epidemic must be considered when recruiting high risk HIV-negative cohorts in prevention research, as demonstrated by the lower than anticipated seroincidence in this study.

Understanding natural history of HIV infection from as early a point following exposure to HIV as possible is key for the design, development and targeting of new prevention interventions as well as for the treatment of advancing HIV disease. Understanding how to identify those most at risk for HIV infection, whether this is due to high-risk behaviours, or due to the increased risk of infection due to high viral loads during acute or recent infection, or due to the increases risk conferred by concomitant sexually transmitted infections, has recently been suggested as the key concern for prevention science [26]. It is hoped that data from the Acute Infection study will contribute significantly to vaccine development by describing the natural history of HIV infection, the impact of host and viral factors during acute infection on viral load set point, as well as the impact of viral set-point on disease progression. Behavioural data collected before and after acute infection should assist in developing algorithms to identify those most at risk of infection. HIV screening algorithms [27] and clinical data collected during acute infection are potentially fruitful explorations of efficient and effective screening algorithms for identifying acute infection. These specific research areas will be addressed separately in publications from the CAPRISA 002 Acute Infection Study Team.
